# Calcium Dynamics in Basal Dendrites of Layer 5A and 5B Pyramidal Neurons Is Tuned to the Cell-Type Specific Physiological Action Potential Discharge

**DOI:** 10.3389/fncel.2017.00194

**Published:** 2017-07-11

**Authors:** Patrik Krieger, Christiaan P. J. de Kock, Andreas Frick

**Affiliations:** ^1^Department of Cell Physiology, Max Planck Institute for Medical Research Heidelberg, Germany; ^2^Department of Systems Neuroscience, Medical Faculty, Ruhr University Bochum Bochum, Germany; ^3^Department of Integrative Neurophysiology, Center for Neurogenomics and Cognitive Research (CNCR), VU University Amsterdam Amsterdam, Netherlands; ^4^INSERM, Neurocentre Magendie, U1215 Bordeaux, France; ^5^University of Bordeaux, Neurocentre Magendie, U1215 Bordeaux, France

**Keywords:** barrel cortex, backpropagating action potentials, calcium, dendrites, active properties, pyramidal neurons, whisker stimulation

## Abstract

Layer 5 (L5) is a major neocortical output layer containing L5A slender-tufted (L5A-st) and L5B thick-tufted (L5B-tt) pyramidal neurons. These neuron types differ in their *in vivo* firing patterns, connectivity and dendritic morphology amongst other features, reflecting their specific functional role within the neocortical circuits. Here, we asked whether the active properties of the basal dendrites that receive the great majority of synaptic inputs within L5 differ between these two pyramidal neuron classes. To quantify their active properties, we measured the efficacy with which action potential (AP) firing patterns backpropagate along the basal dendrites by measuring the accompanying calcium transients using two-photon laser scanning microscopy in rat somatosensory cortex slices. For these measurements we used both “artificial” three-AP patterns and more complex physiological AP patterns that were previously recorded in anesthetized rats in L5A-st and L5B-tt neurons in response to whisker stimulation. We show that AP patterns with relatively few APs (3APs) evoke a calcium response in L5B-tt, but not L5A-st, that is dependent on the temporal pattern of the three APs. With more complex *in vivo* recorded AP patterns, the average calcium response was similar in the proximal dendrites but with a decay along dendrites (measured up to 100 μm) of L5B-tt but not L5A-st neurons. Interestingly however, the whisker evoked AP patterns—although very different for the two cell types—evoke similar calcium responses. In conclusion, although the effectiveness with which different AP patterns evoke calcium transients vary between L5A-st and L5B-tt cell, the calcium influx appears to be tuned such that whisker-evoked calcium transients are within the same dynamic range for both cell types.

## Introduction

In many CNS neurons, including neocortical pyramidal neurons, action potentials (APs) initiated at the axon hillock actively propagate back into the dendrites (Stuart and Sakmann, 1994; Larkum and Zhu, 2002; Waters et al., 2003; Bereshpolova et al., 2007). In addition to providing an important voltage signal, these back-propagating APs (bAPs) can activate calcium channels and elicit a calcium influx in the dendrites and dendritic spines, which triggers numerous intracellular signaling pathways required for the regulation of neuronal function and plasticity (Helmchen et al., 1996; Higley and Sabatini, 2008, 2012; Krieger, 2009; Grewe et al., 2010; Xu et al., 2012; Hill et al., 2013). bAPs have been suggested to provide a crucial signal for the induction of synaptic and non-synaptic forms of plasticity, to lower the threshold for the initiation of dendritic spikes or to trigger dendritic spikes when evoked as trains above a well-defined critical frequency, and to cause the release of transmitters/neuromodulators from the dendrites (reviewed Frick and Johnston, 2005; Dan and Poo, 2006; Sjöström et al., 2008). As such, the regulation of dendritic AP backpropagation efficacy has important implications for neuronal function and plasticity.

In neocortical pyramidal neurons, bAPs—despite being actively supported—decline in amplitude along the dendrites and eventually propagate in a passive manner (Antic, 2003; Kampa and Stuart, 2006; Nevian et al., 2007). The efficacy of AP back-propagation is strongly determined by the intrinsic excitability of the dendrites due to the expression levels of various voltage-gated ion channel (VGIC) types in their membrane. These VGIC distribution patterns are characteristic for particular neuron types, as well as for the different dendritic compartments within single neurons (e.g., the basal dendrites, apical trunk or apical tuft dendrites; Major et al., 2013). To exemplify how this variability affects dendritic function we compared the two major output neuron types of layer 5 (L5) of the somatosensory neocortex—namely L5A slender-tufted (L5A-st) and L5B thick-tufted (L5B-tt) pyramidal neurons. The specific functional role of these neurons within the neocortical circuits is reflected by their differences in dendritic morphology, physiology and connectivity (Manns et al., 2004; de Kock et al., 2007; Groh et al., 2010). For example, L5A-st and L5B-tt cells have different sensory-evoked AP response patterns. In anesthetized rats, L5A-st neurons in the barrel cortex (BC) respond to principal whisker stimulation with on average 0.15 APs per whisker stimulus, whereas L5B-tt cells fire more complex AP patterns with on average 0.64 APs (de Kock et al., 2007). Differences in the morphological properties of basal dendrites between these two neuron types—such as dendritic length or branching pattern (Oberlaender et al., 2012; Narayanan et al., 2015)—could have important consequences for dendritic function. For example, dendritic length affects the efficacy of AP backpropagation and thus the amplitude of their associated calcium transients. Branch points, in turn, may cause propagation failures (Goldstein and Rall, 1974; Stuart et al., 1997b; Vetter et al., 2001). In the present study, we asked how the morphology and active properties of the basal dendrites of the two major classes of L5 pyramidal neurons determine the processing of cell-type specific physiological AP patterns in these dendritic compartments.

To investigate the relationship between physiological AP activity and dendritic function for L5A-st and L5B-tt pyramidal cells in greater detail, we measured bAP-evoked calcium transients in the basal dendrites, in response to a variety of bAP patterns. We found that “artificial” and physiological AP patterns evoked different, yet cell-type specific, calcium profiles in these dendrites. These findings raise intriguing questions regarding the way information is processed in a context-specific manner in these two major output neuron types of the neocortex.

## Materials and Methods

### Slice Preparation and Solutions

Male Wistar rats (26–36 days old) were anesthetized using isoflurane, decapitated and coronal slices (350 μm thick) were prepared from the whisker-related barrel field of the somatosensory cortex (BC). All experimental procedures were carried out according to the animal welfare guidelines of the Max-Planck Society. Brain slices were incubated for 20–30 min at 36°C and then stored at room temperature until used. The extracellular solution for cutting and incubation contained (in mM): 125 NaCl, 25 NaHCO_3_, 2.5 KCl, 1.25 NaH_2_PO_4_, 6 MgCl_2_, 1 CaCl_2_, 3 myo-inositol, 2 Na-pyruvate, 0.4 ascorbic acid, 25 glucose. For the experiments, brain slices were transferred to the recording chamber and superfused with an extracellular recording solution that contained 125 mM NaCl, 25 mM NaHCO_3_, 2.5 mM KCl, 1.25 mM NaH_2_PO_4_, 1 mM MgCl_2_, 2 mM CaCl_2_, 25 mM glucose, and was saturated with 95% O_2_/5% CO_2_ (pH 7.4). Recordings were made at near-physiological temperature of 32–36°C.

### Cell Identification and Electrophysiology

L5A and L5B of the BC were visualized at low magnification (5×) under bright-field illumination, and the pyramidal neurons were visualized with infrared gradient contrast microscopy using a Leica upright microscope fitted with a 40×/0.8 numerical aperture water-immersion objective. In addition to their layer location, L5A-st and L5B-tt pyramidal neurons were selected based on the size of their cell body and apical dendritic trunk (L5A-st: soma diameter 17 ± 3 μm, apical dendrite diameter 3 ± 0.7 μm, *n* = 6; L5B-tt: soma diameter 21 ± 2.8 μm, apical dendrite diameter 5 ± 0.9, *n* = 8; de Kock et al., 2007). Recording pipettes (4–6 MΩ) were pulled from borosilicate glass and filled with (in mM): 135 K-gluconate, 10 HEPES, 10 Phosphocreatine-Na, 4 KCl, 4 ATP-Mg, 0.3 Na-GTP, pH 7.2 (adjusted with KOH). Signals were recorded using an Axon Instrument amplifier (Axoclamp-2B), low-pass filtered at 3 kHz and sampled at 10–50 kHz.

### Stimulation Protocols

Physiological stimulation protocols were designed according to previously recorded trains of APs that were evoked by principle whisker stimulation in L5A-st (84 AP patterns, including 29 patterns where no spikes were elicited, *n* = 6) and L5B-tt (156 AP patterns, including 18 patterns with no APs, *n* = 6) pyramidal neurons in the BC of anesthetized rats (P25–30; de Kock et al., 2007). These 500 ms-long traces are comprised of spontaneous AP output and evoked AP firing (200 ms-long whisker stimulation between 145 ms and 345 ms, for details see de Kock et al. (2007). In this study, we randomly selected 12 different *in vivo* AP patterns for each recorded L5A-st (from a total of 84 patterns) and each L5B-tt (from a total of 156 patterns) cell (Figure 2). To better simulate the *in vivo* situation we included in the random selection also those trains that did not elicit an AP response. The data shown in Figures 2E–H, is thus the average calcium response that could naturally occur during a 500 ms period where spiking could range from zero to several spikes (in our data set: L5-st max 5 spikes; L5B max 11 spikes). The time point of each AP during these 500 ms sequences was taken and a replica trace consisting of 3 ms-long current pulses at the respective time points was used to stimulate the neurons. These “physiological” firing patterns were compared to “artificial” trains of three APs, where the interval between the first and third AP was fixed (50 ms) and the position of the second AP was shifted to occur at 5 ms, 25 ms or 45 ms after the first AP. The second AP was thus shifted to evoke either an AP burst early in the spike train (2nd AP at 5 ms) or at the end (2nd AP at 45 ms). This can be compared with the “physiological” AP pattern where the median interspike-interval (ISI), measured for the first three APs in the train, was for L5A-st 16.1 ms and for L5B-tt 12.9 ms. Furthermore, the L5B-tt patterns had 9% high-frequency-ISIs (defined as ISI ≤5 ms), whereas L5A-st patterns had significantly fewer (1%; Fisher’s exact test, *P* = 0.0091).

**Figure 1:**
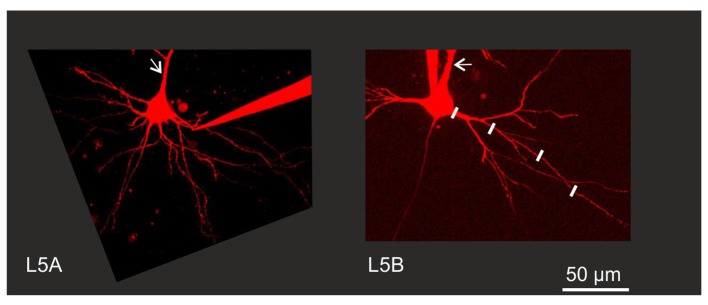
Experimental configuration. Neurons were filled with Alexa 594 to permit visualization of fine morphological details. Back-propagating action potential (BAP) evoked calcium responses were measured in line scan mode at various increments along the dendrite as illustrated by the white lines along one branch of a Layer 5B thick-tufted (L5B-tt) cell basal dendrite. Arrows indicate the apical dendrite. Note the thinner apical and basal dendrites close to the soma in the L5A slender-tufted (L5A-st) cell.

**Figure 2:**
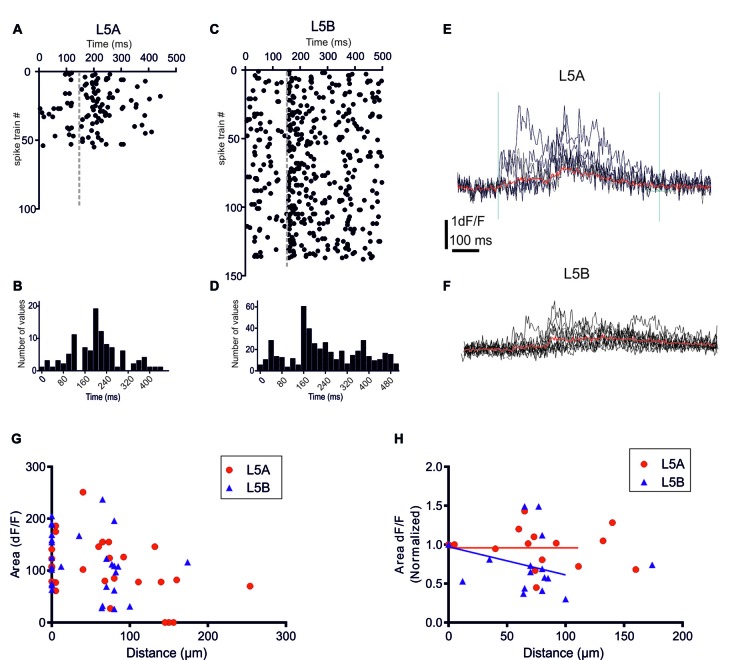
bAP-evoked calcium responses evoked by physiological spike trains action potential (AP) patterns. **(A)** A raster plot showing 55 different *in vivo* recorded spike trains recorded from six L5A-st pyramidal cells. **(C)** The corresponding 138 spike trains recorded from six L5B-tt pyramidal cells. **(B,D)** Summary histograms showing the temporal structure of the L5A-st and L5B-tt spike trains, respectively. **(E)** An example of the bAP-evoked calcium response evoked in a L5A-st cell by seven different *in vivo* recorded spike trains. As illustrated by the two vertical lines (left line marks the time of the first AP in the spike train, and the right line is at +600 ms) the calcium response was quantified by calculating the integral of the d*F*/*F* (*t*) function with *t* = 600 ms. The red sweep shows the average response. **(F)** An example of the bAP-evoked calcium response evoked in a L5B-tt cell (at 35 μm) by 10 different *in vivo* recorded spike trains. The red sweep shows the average response. **(G)** The average calcium response evoked by *in vivo* AP pattern stimulations. The bAP-evoked calcium response is plotted as a function of distance along the basal dendrite. **(H)** The bAP-evoked response normalized separately for each branch to the response at the proximal dendrite location (distance <10 μm). Linear regression (for points up to 125 μm) shows that for L5B-tt (*P* = 0.0412) but not for L5A-st (*P* = 0.3257) the slope deviates from zero, thus suggesting that in L5B-tt, but not in L5A-st the bAP-evoked response decreases at distal points (up to 125 μm).

### Calcium Imaging

**Figure 3:**
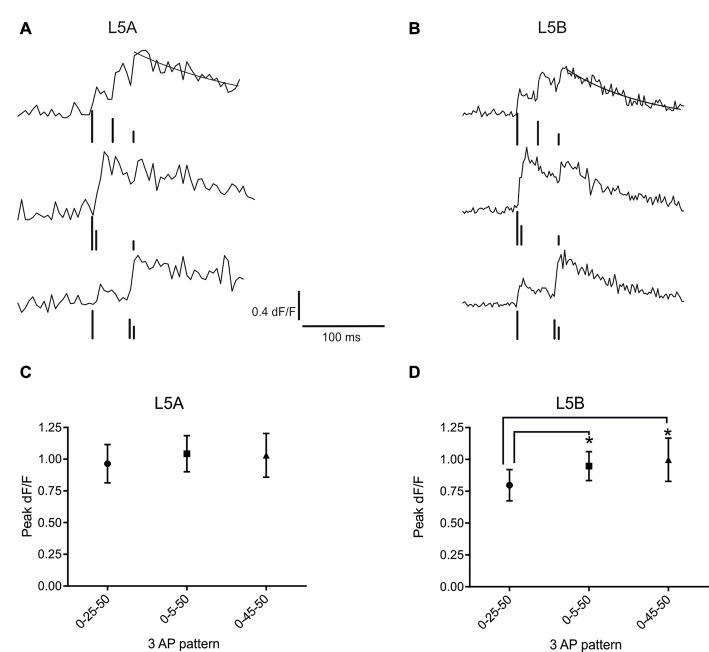
bAP evoked calcium responses induced by artificial firing AP patterns. **(A)** An example of the bAP-evoked calcium responses evoked by the three different 3AP patterns in a L5A-st cell (measured at 68 μm from the soma). The peak calcium response was calculated by fitting a standard exponential to the decay phase (black line). The black bars illustrate the time point of AP stimulation. The upper trace corresponds to the 0–25–50 ms stimulus, middle trace: 0–5–50 ms and the lower trace: 0–45–50 ms. The vertical lines are drawn at different heights, for the purpose of clarity. **(B)** Representative example of the bAP-evoked calcium responses measured in a L5B-tt cell (154 μm from the soma) and induced by the same three 3AP patterns used in **(A)**. Analyzing the three different 3AP patterns separately shows that in L5A cells **(C)** there was no effect of a high frequency component (200 Hz interspike interval, ISI) in the 3AP trains (averaged from dendritic spots at 40–254 μm), whereas in L5B cells **(D)** the two 3AP patterns with a high frequency component evoked a larger calcium response compared to the 3AP pattern with a 40 Hz ISI (averaged from dendritic spots at 57–168 μm). *P < 0.05.

**Figure 4:**
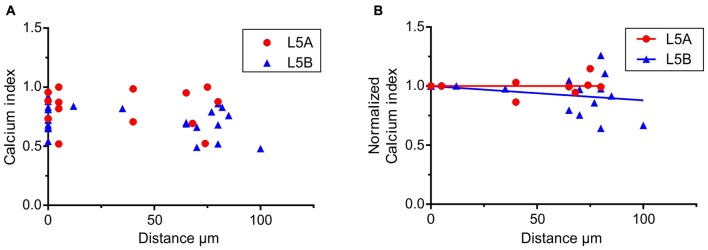
Calcium responses elicited by simulated whisker-evoked AP patterns. The aim in this analysis was to quantify the relative changes in calcium caused by whisker stimulation. The larger the post-whisker stimulation evoked calcium influx is in comparison to the calcium influx before whisker stimulation, the closer the calcium index [1 – (Ca-pre-whisk/Ca-post-whisk)] is to one. **(A)** In both L5A-st and L5B-tt cells the calcium response increases due to whisker deflection (the calcium index is >0). **(B)** To quantify the changes along the length of the basal dendrite the bAP evoked calcium response was normalized to the response close to the soma (5–10 μm) for each individual dendrite branch. In L5A-st cells the calcium index remains close to 1 (linear regression *P* = 0.7804) through the length of the basal dendrite (measured up to 125 μm), indicating that there is a substantial relative increase in calcium after whisker stimulation. In contrast in L5B-tt cells the calcium index decreases slightly with distance (linear regression, *P* = 0.0204) indicating that further out on the basal dendrite the relative increase in calcium influx caused by whisker stimulation decreases.

The method used for high-resolution, multi-photon imaging of Ca^2+^ fluorescence was as described previously (Krieger, 2009; Grewe et al., 2010). In brief, one system was a Leica TCS-SP2RS scanning unit attached to an upright Leica microscope (DMLFS) and equipped with a 40× objective (HCX APO, 0.8 NA, Leica). Two-photon excitation was achieved with a Ti:Sa-laser (MIRA 900F; Coherent, Santa Clara, CA, USA) at 840 nm pumped by a solid-state laser (Verdi 5W; Coherent). A dichroic mirror (560DCXR) split the fluorescence signal into one detector (bandpass filter, HQ525/50M) for green fluorescence (Oregon Green 488 BAPTA-I, calcium signals), and another detector (bandpass filter, HQ610/75M) for red fluorescence (Alexa 594, fluorescent marker for dendritic morphology). The second system was a galvanometer scanning unit (TCSNT; Leica Microsystems, Mannheim, Germany) that was mounted on an upright microscope (Leica DMLF) equipped with a 40× objective (HCX APO W40×/0.8 NA), utilizing a Ti:Sa-Laser (Mira 900F; Coherent, Santa Clara, CA, USA) at 870 nm pumped by a solid-state laser (Verdi 8W; Coherent), and using similar filters for the detection of the green/red fluorescence signals. Calcium signals were recorded in line-scan mode with a temporal resolution of 2–2.2 ms/line, with a total scan time per trial in the range of 256 ms to 2.3 s. Recordings of membrane voltage and fluorescence were analyzed offline using pClamp 9 (Molecular Devices, Sunnyvale, CA, USA). Neurons were filled with a combination of the calcium-sensitive dye Oregon Green 488 BAPTA-I (200 μM) and the calcium-insensitive dye Alexa 594 (20–50 μM; both dyes from Invitrogen, Carlsbad, CA, USA) added to the intracellular recording solution to visualize the basal dendrites and to measure Ca^2+^ signals. To ensure a homogeneous dye distribution within the basal dendrites under investigation, dyes were allowed to fill the neuron for at least 20 min before Ca^2+^ signals were measured. Ca^2+^ transients are reported as relative changes in OGB-1 fluorescence and were calculated as d*F*/*F* (*t*) = (*F*(*t*) − *F*0)/(*F*0 − *F*B). Background fluorescence (*F*B) was measured in a neighboring indicator-free region. Resting fluorescence (*F*0) was calculated as the average value of the 50–100 ms period that preceded the stimulation. Calcium transients associated with physiological AP patterns (Figures 2A,C) were quantified by calculating the calcium-time integral during a 600 ms period starting from the 1st AP in the train (Figure 2E). The area rather than the peak amplitude was calculated since the goal was to measure the total calcium response at a given dendritic spot during the whole stimulation epoch. For simulated whisker-evoked calcium responses (Figure 4) the area was measured 145 ms before and 145 ms after the time point when, during the *in vivo* recordings (de Kock et al., 2007), the whisker was deflected. The peak amplitude of the calcium response evoked by the artificial 3AP patterns was quantified by a single exponential fit to the decay of the fluorescence transients, calculated as the averages of 3–5 trials. In the normalized plots (Figures 2H, 4B) the null hypothesis (tested using nonlinear regression analysis to fit a straight line) was that the calcium response did not change with distance, thus that the slope was zero. The comparison of the effect of the three different 3AP patterns (Figures 3C,D) was performed with repeated measures ANOVA, thus at each dendritic spot the three 3AP patterns were compared to each other. Data are presented as mean ± SD. GraphPad Prism 6 (GraphPad Software, San Diego, CA, USA) was used for statistical analysis. The diameter of the basal dendrites was obtained from the two-photon microscopy images.

## Results

The basal dendrites of L5 pyramidal neurons receive the vast majority of synaptic inputs within L5 and are thus crucial for intracortical communication (Frick et al., 2008). L5 contains two main types of pyramidal neurons, but whether or not their basal dendrites have different functional properties has not directly been addressed. We therefore compared the properties of basal dendrites for L5A-st and L5B-tt neurons by measuring their backpropagation efficacy of cell-type specific, physiological AP patterns. These AP trains were triggered by somatic current injections and the accompanying calcium transients were measured along the basal dendrites using two-photon laser scanning microscopy as illustrated in Figure 1. L5A-st and L5B-tt neurons were selected based on their layer location, cell body diameter and size of the apical dendritic trunk (see also “Materials and Methods” Section).

### Dendritic Calcium Responses Evoked by Physiological AP Patterns

Previously, we demonstrated that in L5A-st pyramidal neurons the temporal structure of physiological AP patterns is an important determinant for their backpropagation efficacy along apical but not basal dendrites (Grewe et al., 2010). We now addressed this issue comparing with the basal dendrites of L5B-tt neurons. *In vivo* recordings from L5A-st and L5B-tt pyramidal neurons of anesthetized rats have demonstrated significantly different spontaneous and evoked AP firing patterns in these neuron types (Manns et al., 2004; de Kock et al., 2007; de Kock and Sakmann, 2008, 2009). The randomly selected physiological AP firing patterns from L5A-st pyramidal neurons used in this study contained 1–5 APs (median: 1AP/500 ms; (Figures 2A,B), while the sequences recorded from L5B-tt pyramidal neurons consisted of 1–11 APs (median: 3APs/500 ms; Figures 2C,D). The bAP-evoked calcium responses were measured in the proximal region of the basal dendrite (5–10 μm from soma) and at different more distal locations along the same dendrite (40–278 μm). Even though on average more APs were evoked in L5B-tt neurons compared to L5A-st neurons, the average bAP-evoked calcium response evoked by a series of physiological AP patterns appeared to be similar in L5A-st and L5B-tt cells (Figure 2G) with regard to the absolute magnitude (proximal location: L5A 113 ± 49 (unit: time × d*F*/*F*), *n* = 7 dendrite spots (branches), 7 cells; L5B 131 ± 50, 13 dendrite branches, 10 cells, *P* = 0.4519, unpaired *t*-test; distal locations: L5A 106 ± 61, *n* = 10 dendritic spots, 5 cells; L5B 98 ± 61, 12 dendritic spots, 10 cells, *P* = 0.7646, unpaired *t*-test). Although averaging over all cells within the population can show general trends, the large variability between cells and dendrite branches from the same cells argues that a normalization for each branch (as in Figure 2H) is more representative of how the bAP evoked calcium response changes along the basal dendrite. Normalizing to each measured dendritic branch (Figure 2H), the calcium response measured at the proximal site for that branch, suggests that in L5B-tt (linear regression *P* = 0.0011), but not in L5A-st neurons (linear regression, *P* = 0.2332), there is a small decrease with distance along the dendrite (dendritic sites 0–125 μm). This difference in the calcium response along the dendrite (0–125 μm) between L5A-st and L5B-tt neurons is corroborated by a two-way ANOVA analysis of the average data (Figure 2G), which for the Interaction effect has a *P* value of 0.05.

### bAP-Induced Calcium Responses Evoked by High-Frequency AP Patterns

Next, we asked whether the presence of a high frequency component would alter the efficacy with which the bAPs can evoke a calcium response. For this purpose, trains of three APs were used, where the interval between the first and third AP was fixed and the position of the second AP varied. One AP train had equally spaced APs (occurring at 0, 25 and 50 ms) at sub-critical frequency (40 Hz, mean-frequency pattern, *F*_mean_; Kampa and Stuart, 2006), while the other two trains contained a supra-critical frequency (200 Hz) component positioned either at the beginning (0–5–50 ms; High-Low-frequency pattern, *F*_H–L_) or the end (0–45–50 ms; Low-High-frequency pattern, *F*_L–H_) of the train. Representative examples of the calcium transients evoked by these AP patterns at a single position in the basal dendrites of L5A-st and L5B-tt neurons are shown in Figures 3A,B, respectively. In L5A-st basal dendrites (range: 40–254 μm; median: 96 μm), back-propagation resulted in a similar calcium response for all three AP patterns (*F*_mean_: 0.96 ± 0.58% d*F*/*F*; *F*_H–L_: 1.04 ± 0.55% d*F*/*F*; *F*_L–H_: 1.03 ± 0.67% d*F*/*F*; 8 cells, 15 dendritic positions *P* = 0.3253, repeated measure ANOVA; Figure 3C). In contrast, in L5B-tt pyramidal neurons (range: 57–168 μm; median: 101 μm) the amplitude of the calcium signal was significantly different for the three AP patterns (Figure 3D). The presence of a high-frequency component (*F*_H–L_ and *F*_L–H_ pattern) produced ~20% larger transients compared to the *F*_mean_ pattern (*F*_mean_: 0.80 ± 0.41% d*F*/*F*; *F*_H–L_: 0.95 ± 0.38% d*F*/*F*; *F*_L–H_: 1.00 ± 0.56% d*F*/*F*; *P* = 0.0130 with repeated ANOVA and Holm-Sidak’s multiple comparisons test; *F*_mean_ vs. *F*_H–L_, *P* < 0.05; *F*_mean_ vs. *F*_L–H_ *P* < 0.005, *n* = 7 cells, 11 dendritic positions). Taken together, these data suggest significant differences in the intrinsic properties of the basal dendrites of these two neuron populations, affecting the processing and propagation of information.

The physiological firing patterns derived from L5A-st neurons typically involve fewer APs than those derived from L5B-tt neurons (Figures 2A,B), thus the relatively larger calcium response in L5A-st suggest that L5A-st basal dendrites respond with a larger calcium transient per AP. To examine this point in greater detail, we used identical (“artificial”) 3AP patterns for both cell types to evoke calcium transients measured in a proximal location (5–10 μm from soma periphery). In agreement with the results using the physiological firing patterns, we found that when stimulated with identical 3AP patterns, the average calcium response (the average of all three different AP patterns), was significantly larger in L5A-st basal dendrites (1.29 ± 0.48% d*F*/*F*, unpaired *t*-test, *P* = 0.0083, *n* = 10 cells, data not shown) compared to L5B-tt basal dendrites (0.71 ± 0.13% d*F*/*F*, *n* = 7 cells). One possible explanation for these findings is related to the difference in diameter of basal dendrites at this proximal location for both neuron types. We found that the diameter of L5A-st proximal basal dendrites (≤10 μm from soma) was significantly smaller compared to L5B-tt basal dendrites (L5A-st: 1.71 ± 0.41 μm, *n* = 12 dendrites; L5B-tt: 2.32 ± 0.59 μm, *n* = 10 dendrites; unpaired *t*-test, *P* = 0.0104). A smaller diameter and therefore an increased surface-to-volume ratio may explain the larger calcium response in L5A-st compared to L5B-tt neurons (Holthoff et al., 2002; Anwar et al., 2014). At more distal dendritic locations there was no difference in diameter (L5A-st: 0.78 ± 0.15 μm, *n* = 18, median distance from soma 95 μm, range: 40–170 μm; L5B-tt: 0.80 ± 0.22 μm, *n* = 21, median distance from soma 100 μm, range: 20–168 μm; unpaired *t*-test, *P* = 0.7169).

### Calcium Responses Induced by Simulated Whisker-Evoked AP Patterns

To further investigate how calcium responses are evoked by natural stimulations, we specifically looked at the calcium responses elicited by the APs evoked by whisker stimulation. In the 500 ms long recordings used as a template for the firing patterns, whisker stimulation occurred at 145 ms (see Figures 2A,C). Under physiological conditions spontaneous spikes also back-propagate and are thus capable of eliciting calcium responses. Taking this into account, we calculated the relative increase in calcium before and after the simulated whisker-evoked AP pattern. The relative increase should better reflect the changes in calcium specifically induced by whisker stimulation. To evaluate the calcium response elicited by APs evoked by a “whisker-like” stimulation, we thus compared the calcium response elicited by the AP pattern during a time window of 145 ms before (spontaneous activity; “Ca-pre-whisk”) and after the simulated “whisker” stimulus (spontaneous plus evoked activity; “Ca-post-whisk”). Calcium responses were measured as previously described (see Figure 2 and “Materials and Methods” Section), with the difference that now only 145 ms was used to calculate the calcium response (the calcium-time integral). In the physiological AP pattern used in this part of the study the average number of APs during the “pre-whisk” stimulation period was for L5A-st 0.29 APs/145 ms and during “post-whisk” 0.64 APs/145 ms, and for L5B-tt 0.53 and 1.11 APs/145 ms, respectively. Calcium responses were normalized with respect to a “calcium index” calculated as 1 − (Ca-pre-whisk/Ca-post-whisk). The closer the calcium index is to 1 the larger the relative increase in calcium evoked by the simulated whisker-evoked AP pattern (Figure 4A). Measuring the relative change in calcium is important because, presumably, not only the absolute calcium amplitude but also the “whisker-evoked” change is crucial for dendritic computation. Normalizing the calcium index to the value at the dendrite spot close to the soma (5–10 μm), for each basal dendrite separately (L5A-st 7 cells, 7 basal dendrites; L5B-tt: 8 cells, 11 basal dendrites), indicates (Figure 4B) that with distance along the basal dendrite the calcium index decreases for L5B-tt cells (linear regression *P* = 0.0204) but not for L5A-st cells (linear regression *P* = 0.7804). A decreased index would thus suggest that the relative increase in calcium influx as a result of whisker stimulation is smaller in L5B-tt than in L5A-st. Presumably this is caused by the higher spontaneous discharge, (see Figure 2C) in the physiological patterns used to mimic L5B-tt cell activity, such that any additional APs evoked by the whisker stimulation has a relatively smaller effect. In comparison the L5A-st cells had a lower spontaneous firing rate in the physiological patterns used, even sometimes having no spikes preceding the whisker stimulus at 145 ms (see Figure 2A). In summary our data show that because whisker evoked spike trains differ between L5A-st and L5B-tt neurons the physiological effect on calcium dynamics is such that the basal dendrites are optimized to respond to different features of the spike trains. While L5A-st basal dendrites are more responsive to an increase in firing, the L5B-tt basal dendrites are in addition also responsive to the presence of high frequency spikes.

## Discussion

Here we examined information processing in the basal dendrites of L5 neurons of the somatosensory cortex. Specifically, we examined how one important aspect of information processing—AP backpropagation—differs between the two populations of principal neurons of this layer. We show that in spite of the marked differences in the nature of recorded physiological AP patterns the basal dendrites respond to these natural stimuli with fairly similar calcium responses in proximal dendrites (<10 μm from soma; Figure 2). However, when exposed to identical patterns of stimulation, in the form of artificial 3AP trains, the average calcium signal elicited in proximal dendritic locations is larger in L5A-st than in L5B-tt basal dendrites (Figure 3). A further discrepancy was that the efficacy with which physiological AP patterns evoked a calcium response appeared to decrease in L5B-tt but not in L5A-st (Figure 2H). It is furthermore apparent that the calcium profile along the dendrite is dependent on the specific AP pattern used to evoke a calcium transient. When using the same AP pattern, the 3AP pattern, to evoke calcium transients in both cell types, the data indicates that in the proximal dendrite the calcium response is larger in L5A-st cells. Using a physiological AP pattern, we show that on average the calcium responses evoked during physiological conditions appear to be similar for L5A-st and L5B-tt in a proximal dendrite, but for L5B-tt cells there is a decrease in the calcium evoked transient in the more distal parts of the basal dendrite. The finding that a high-frequency component can cause a boosting of the calcium response in L5B-tt cells is interesting since burst spiking in L5B-tt cells may be crucial for sensory perception (Takahashi et al., 2016). The apparent difference between L5A-st and L5B-tt cells in calcium responses elicited by the same “artificial” standardized 3AP patterns thus suggests that dendritic properties determining calcium influx differ for L5A-st and L5B-tt cells. However, when the dendrites process physiological AP patterns, with more APs in the L5B-patterns, both cell types still show only a relatively minor difference in the calcium response profile. This could indicate that although the output activity, and thus the AP patterns that back-propagate into the dendrites, are very different in L5A-st and L5B-tt neurons, the dendrites are tuned in such a way that the efficacy with which physiological trains of APs propagate is relatively similar.

The morphology of dendrites has a strong impact on the active properties of neurons (Vetter et al., 2001; Schaefer et al., 2003). The most obvious morphological difference between L5A-st and L5B-tt neurons is the architecture of the apical dendrite, but cell-type specific differences in the basal dendrites have also been observed (Narayanan et al., 2015). For instance, dendritic length, volume, horizontal span and number of branch points are significantly larger (all comparisons *P* < 0.01) for basal dendrites of L5B-tt neurons compared to basal dendrites of L5A-st neurons (Narayanan et al., 2015). In the present study, we also show that the diameter of basal dendrites is cell-type specific (see “Results” Section) and these morphological differences impact propagation of electrical signals in these dendrites. A thinner L5A-st basal dendrite close to the soma will contribute to the larger calcium response in comparison to the thicker L5B-tt basal dendrite. A calcium increase in L5B-tt cells could thus be partially explained by the reduction in the diameter of the dendrite along its proximal-distal axis. The majority of synapses on the basal dendrites of the L5 pyramidal cells are located in the range from 50 μm to 150 μm from the soma (Frick et al., 2008). We hypothesize that this is due to a tuning mechanism, which maintains the calcium levels in the two cell types within the same dynamic range. It remains to be investigated if this gradient, in the absolute calcium concentration, along the dendrite affects synaptic integration at different dendritic spots, and how different spike trains are encoded by a difference in the calcium response. A further methodological consideration is that while we analyzed the total calcium response (measuring the area), other parameters such as the timing of the peaks of the calcium transients may also be important.

The dendritic back-propagation efficacy of AP trains typically differs from that of single APs, due to the biophysical properties of the dendritic VGICs that are activated under those firing regimes. The number of APs and their instantaneous intervals (i.e., frequencies) define the temporal structure of the AP trains, and are therefore the main parameters determining their efficacy (Spruston et al., 1995; Stuart et al., 1997a; Larkum et al., 1999; Williams and Stuart, 2000). Further investigation is necessary to determine how the temporal bAP-pattern (Gütig and Sompolinsky, 2006) can be encoded in the calcium response.

The L5A-st and the L5B-tt basal dendrites thus appear to be tuned to optimally respond to information of a certain quality, i.e., responding to different AP input patterns. For example, high frequency AP firing is more efficiently transmitted along the dendrite in L5B-tt compared to L5A-st neurons. This observation may be explained by differences in the cable properties of these two populations of neurons. It may also be explained by differences in the gradients of ion channels along the dendritic length. These possible mechanisms remain to be investigated further. Lastly, we emphasize the importance of choosing appropriate stimulation protocols for analyzing how backpropagating AP patterns are encoded in dendritic calcium responses.

### Comparison with Back-Propagation in Apical Dendrites and during Postnatal Development

Previous studies have shown that bAP-evoked calcium responses differ in apical dendrites in L5A-st and L5B-tt pyramidal cells. For example, the calcium response to high-frequency AP patterns was more strongly attenuated in L5A than in L5B apical dendrites (Schiller et al., 1995; Grewe et al., 2010). Some aspects of the efficacy of bAP propagation has previously been reported for L5A pyramidal neurons (Grewe et al., 2010). In reference to that study the following interesting comparison can be made. It was shown that in the apical dendrite there is a developmental change between P14 and P25 such that in older animals the response to a 3AP pattern initially increases rather than decreases (see Figure 7B in Grewe et al., 2010). A similar developmental change is evident for the L5A basal dendrites where at P14 there is a marked decrease along the length of the dendrite (Figure 6C in Grewe et al., 2010) which is not the case at the older ages reported in the present study.

### Whisker Evoked Calcium Response

Using a variety of artificial 3AP patterns we show that L5A-st and L5B-tt pyramidal cells have different calcium responses depending on the exact temporal structure. These differences are likely to be important for synapse specific differences in synaptic plasticity. Interestingly, however, the AP pattern evoked by simulated whisker stimulation elicited rather similar calcium responses in both cell types even though the temporal structure and number of APs in the trains used differed. In our calculation we analyzed the relative increase in calcium evoked by APs occurring before and after whisker stimulation (thus providing a “calcium index”). The calculation depends not only on the whisker evoked AP patterns but also the spontaneous activity. Considering that L5B-tt neurons fire more APs it is somewhat surprising that the ratio post/pre is not larger in L5B-tt compared to L5A-st dendrites. This could be explained by the fact that L5A-st neurons fire few spontaneous spikes so whenever there is a spike there is a large influx in calcium.

The evidence presented in this *in vitro* study suggesting variability in the calcium responses evoked by AP patterns can be due to a variety of factors, including morphology and channel density. *In vivo* there is also likely a contribution of neuromodulatory states and activity-dependent plasticity (Hoffman and Johnston, 1998; Golding et al., 2001; Frick et al., 2004). In summary the data show that: (1) high frequency AP patterns are more efficiently propagated in L5B-tt compared to L5A-st neurons (Figures 3C,D); (2) the calcium response gradient is such that *in vivo* evoked AP patterns are more strongly attenuated in L5B-tt neurons (Figure 2H); (3) in distal locations the absolute calcium increase is similar in L5A-st and L5B-tt basal dendrites, even though the calcium responses are evoked by very different AP patterns. Considering that L5A-st and L5B-tt pyramidal cells receive input from different part of thalamus, and project to different sub-cortical structures (Groh et al., 2010) it is of interest to investigate how dendritic calcium dynamics is related to functional differences. Whisker evoked responses in ventral posterior medial thalamic nucleus (VPM) are larger (more spikes per stimulus) compared to responses in the posterior medial thalamic nucleus (POm; Diamond et al., 1992). Interestingly VPM axons target L5B and POm targets L5A, thus the higher sensitivity of L5B-tt neurons to high frequency AP firing could be related to the fact that high frequency bAP can be timed to higher frequency inputs and thus facilitate dendritic integration. The less active POm cells can, however, still activate a rather large calcium response in L5A-st cells because the L5A-st cells are “tuned” to integrating lower frequency inputs. Future experiments using physiological VPM/POm spike patterns to create dendritic stimulations in combination with physiological bAP patterns would be used to further investigate this question.

## Author Contributions

PK, CPJK and AF designed the experiments; wrote the article. PK and AF performed and analyzed the *in vitro* experiments.

## Conflict of Interest Statement

The authors declare that the research was conducted in the absence of any commercial or financial relationships that could be construed as a potential conflict of interest.
